# RelB regulates Bcl-xl expression and the irradiation-induced apoptosis of murine prostate cancer cells

**DOI:** 10.3892/br.2014.250

**Published:** 2014-03-12

**Authors:** LIANG ZHU, BIN ZHU, LUOYAN YANG, XIAOKUN ZHAO, HONHYI JIANG, FANG MA

**Affiliations:** 1 Department of Urology, Second Xiangya Hospital, Central South University, Changsha, Hunan 410011, P.R. China; 2 Department of Oncology, Second Xiangya Hospital, Central South University, Changsha, Hunan 410011, P.R. China

**Keywords:** RNA interference, RelB, Bcl-xl, radiosensitivity, prostate cancer

## Abstract

Apoptosis in prostate cancer (PCa) induced by ionizing radiation (IR) is believed to play a critical role in radioresistance. Bcl-xl, an important member of the anti-apoptotic Bcl-2 family, has critical roles in tumor progression and development. The aim of the present study was to investigate the association of Bcl-xl expression and radiosensitivity from murine PCa RM-1 cells. An adenovirus-mediated RNA interference technique was employed to inhibit the expression of the RelB gene. RelB proteins were detected upon irradiation following transfection with small interfering (si)RelB, as shown by western blot analysis. The radiosensitivity of the RM-1 cells was determined by clonogenic assays. The apoptosis of the RM-1 cells were detected by flow cytometry assay, then quantitative polymerase chain reaction assays were performed to determine the expression level of Bcl-xl mRNA in the RM-1 cells. Radiation treatment increased the RelB protein levels from the cytosol and nucleus in the RM-1 cells. The protein expression levels of RelB in the pLentilox-sh-RelB-transfected RM-1 cells were significantly lower than in the negative interference group following radiation treatment. The percentage of cells undergoing apoptosis in the siRelB-RM-1 group was significantly higher than that in the control group following radiation treatment. Finally, a positive link between Bcl-xl expression and RelB activity was established in the RM-1 cells. Inhibition of RelB correlates with a decrease in expression of Bcl-xl. In conclusion, adenovirus-mediated siRNA targeting RelB inhibits Bcl-xl expression, enhances radiosensitivity and regulates the irradiation-induced apoptosis of the murine PCa RM-1 cell line.

## Introduction

Prostate cancer (PCa) is the leading cause of new cancer cases and the second most common cause of cancer-related mortality in men in the US (1). Radiotherapy is commonly employed as curative therapy for locally confined PCa and is also used for salvage therapy in individuals who have undergone a failed radical prostatectomy. However, radiorecurrent PCa and a poor long-term prognosis are experienced by numerous PCa patients, as ~30% of those individuals treated with potentially curative doses develop radioresistance (2). Current strategies employed to improve outcomes include The addition of androgen deprivation therapy (ADT) and radiation dose escalation are two strategies currently being used to improve the outcome for PCa patients. These techniques have successfully been able to reduce the biochemical failure rates. However, even with these improvements, the rates of biochemical failure remain poor for those individuals with higher risk localized and locally advanced diseases (3).

NF-κB plays a significant role in tumorigenesis and resistance to therapy-induced cytotoxicity (4). Thus, inhibition of this protein complex is being contemplated as a target to enhance the efficacy of conventional radiotherapy and chemotherapy (5). In our previous study, it was demonstrated that the RNA interference of RelB could enhance the radiosensitivity of the PCa RM-1 cell line and induce apoptosis (6). Anti-apoptotic Bcl-xl is significant in tumor progression, development and radioresistance (7,8). Ionizing radiation (IR) has previously been shown to activate Bcl-xl in PC-3 cells, and upregulation of this protein results in decreased cell radiosensitivity (9). Thus, the disruption of anti-apoptotic pathways may be a novel target for overcoming radioresistance in PCa. The aims of the present study were to determine if there was an association between the RelB/p52 alternative NF-κB pathway and Bcl-xl in the mouse PCa RM-1 cell line, and to analyze the association between Bcl-xl expression and radiosensitivity in the RM-1 cells.

## Materials and methods

### Cell culture and treatment

RM-1 is an androgen-independent PCa cell line (10), which was purchased from the Cell Culture Collection, Chinese Academy of Sciences (Beijing, China) and maintained in Dulbecco’s modified Eagle’s medium (HyClone™; Thermo Scientific, Logan, UT, USA), supplemented with 10% fetal bovine serum (Gibco, Grand Island, NY, USA). The RM-1 cells were plated in 6-well plates at a density of 5×10^5^ cells/well. A lentiviral vector expressing siRelB (pLentilox-sh-RelB) was constructed, as described previously (11). The transfection efficiency of >95% was used for the subsequent experiments.

### Radiation exposure

The RM-1 cells in the 6-well plates were irradiated via a 6 MeV linear accelerator (Varian Clinac 21EX; Varian Medical Systems, Inc., Palo Alto, CA, USA) at room temperature. Each type of cell received a single dose of 2, 4, 6, or 8 Gy per treatment. Dose rates were given at 2.25 Gy/min. The distance between the radiation source and the cells was 100 cm.

### Clonogenic survival assay

Subsequent to radiation exposure, the RM-1 cells from each well were trypsinized and grown in triplicate in 60-mm culture dishes with different densities (20, 40, 100, 200 and 400 per dish) for 14 days. Once the cells had been fixed in methanol/acetic acid (3:1) for 30 min, the cell clones were counted under a microscope (>50 cells/clone). Plating efficiency (PE) was calculated by dividing the average number of colonies per plate by the amount of cells plated and multiplying by 100. Survival fractions (SFs) were calculated using the formula SF = colony number / (cell number cultured × PE). A one-hit multi-target model was fitted to the cell survival curve [SF = 1 - 1 (1 - e^−KD^)^N^, SF = 1 - (1 - e^−D/D0^)^N^(D_0_ = 1 / K)] to determine the dose quasithreshold (D_q_), the mean lethal dose (D_0_), the 2 Gy SF (SF_2_), the N-value and the sensitization enhancement ratio (SER).

### Measurement of apoptosis by flow cytometry

Once the RM-1 cells had been exposed to radiation at a single dose of 6 Gy, the cells were incubated in normal medium for 48 h. The cells were then collected ([according to the Annexin V-FITC apoptosis detection kit (Byotime, Nantong, China)] and analyzed immediately on a FSCAN flow cytomer (BD Biosciences, Franklin Lakes, NJ USA).

### Western blot analysis

For each treatment group, proteins from the cytosol and nucleus were isolated from the RM-1 cells. subsequent to being blocked in 5% milk for 1 h, the membrane was incubated with the primary antibody, followed by the corresponding secondary antibody. Total proteins (50 μg) were separated using 10% SDS-PAGE and were transferred onto a nitrocellulose membrane. The RelB protein was detected with a primary rabbit RelB monoclonal antibody (1:1,000; Invitrogen Life Technologies, Carlsbad, CA, USA) and a horseradish peroxidase-conjugated anti-rabbit secondary antibody (KPL, Inc., Gaithersburg, MD, USA).

### Quantitative polymerase chain reaction (qPCR)

Total RNA (1 μg) isolated from the RM-1 cells using TRIzol reagent (Invitrogen Life Technologies) was reverse-transcribed using oligo-dT primers with reverse transcription reagents (Toyobo, Co., Ltd., Osaka, Japan). Reverse-transcribed RNA was amplified with SYBR-Green PCR Master mix (Toyobo) plus 0.4 μM of gene-specific upstream and downstream primers during 40 cycles on an Applied Biosystems 7500 Fast Realtime Cycler (Applied Biosystems, Carlsbad, CA, USA). Each cycle consisted of denaturation at 95°C for 15 sec, annealing at 55°C for 15 sec and extension at 72°C for 45 sec. The primer sequences were as follows: β-actin forward, 5′-CCGTGAAAA GATGACCCAG-3′ and reverse, 5′-TAGCCACGCTCGGTC AGG; and Bcl-xl forward, 5′-CCCAGAAAGGATACAGCT GG-3′ and reverse, 5′-GCGATCCGACTCACCAATAC-3′. Data were analyzed by relative quantification using the 2^−ΔΔCt^ method (12).

### Statistical analysis

Statistical analysis was performed using a one-way analysis of variance for multiple group comparisons using SPSS 11.5 software (SPSS, Inc., Chicago, IL, USA). Data are presented as the mean ± standard deviation. P<0.05 was considered to indicate a statistically significant difference.

## Results

### Adenovirus-mediated small interfering RNA (siRNA) targeting RelB inhibits the expression of RelB protein in RM-1 cells

**Figure 1:**
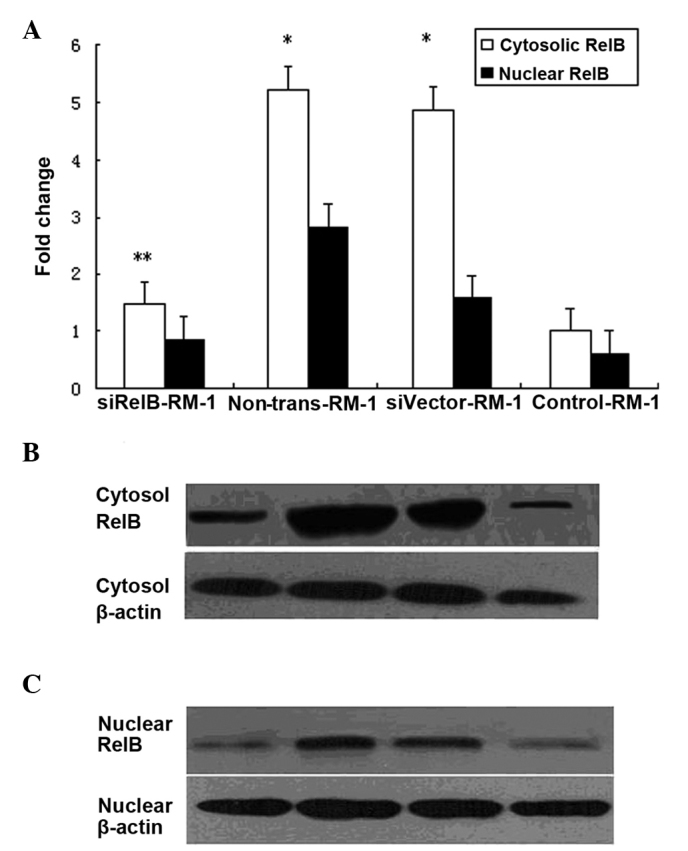
RelB-siRNA decreases RelB protein levels in RM-1 cells following radiation treatment. (A) N-fold changes of RelB mRNA level. Non-trans-RM-1, siVector-RM-1 and siRelB-RM-1 cells were treated by 6 Gy irradiation. (^*^P<0.05 and ^**^P<0.01 vs. control). (B) RelB protein cytosol levels, observed by western blot analysis in response to 6 Gy radiation. (C) RelB protein nuclear levels, observed by western blot analysis, in response to 6 Gy radiation. si, small interfering; siRelB-RM-1, pLentilox-sh-RelB-transfected RM-1 cells; non-trans-RM-1, non-transfected RM-1 cells; siVector-RM-1, RM-1 cells transfected with empty vector; control-RM-1, non-irradiated RM-1 cells.

Western blot analyses were performed to determine the expression level of the RelB protein prior to and following IR in the murine PCa RM-1 cell line. The RM-1 cells transfected with the empty vector (siVector-RM-1) and the non-transfected RM-1 cells (non-trans-RM-1) were treated with a 6 Gy dose. Following treatment, the total RelB proteins from the varying transfected cells were isolated and subjected to western blot analysis using cell lysate from the cytosolic and nuclear fractions. The results showed that the expression levels of RelB protein were markedly higher in the siVector-RM-1 and non-trans-RM-1 cells than in the non-irradiated RM-1 cells (control-RM-1). Compared with the level of the control-RM-1 cells, the RelB protein levels of the non-trans-RM-1 cells were higher by ~5.2- and 2.8-fold in the cytosolic and nuclear fractions, respectively. Similarly, the RelB protein levels of the siVector-RM-1 cells were higher by ~4.9- and 1.6-fold in the cytosolic fractions and nuclear fractions (P<0.05) (Fig. 1). There was no difference in the expression levels of the RelB protein between the non-trans-RM-1 and siVector-RM-1 cells. Thus, it was concluded that IR may promote the expression of RelB in RM-1 cells.

To further analyze the effect of the adenovirus-mediated siRNA-targeting of RelB on the expression of the RelB protein in the RM-1 cell line, pLentilox-sh-RelB-transfected RM-1 cells (siRelB-RM-1) were treated with a 6 Gy dose. The RelB protein levels in the siRelB-RM-1 cells were ~71.8 and 70.4% less in the cytosolic and nuclear fractions compared with that of the non-trans-RM-1 cells (P<0.01). in comparison to the level in the non-trans-RM-1, the RelB protein levels in the siRelB-RM-1 cells exhibited similar manifestations (Fig. 1). These data showed that adenovirus-mediated siRNA targeting RelB could specifically and significantly inhibit the expression of RelB protein in RM-1 cells.

### pLentilox-sh-RelB significantly increases the sensitivity of RM-1 cells to IR in vitro

**Figure 2:**
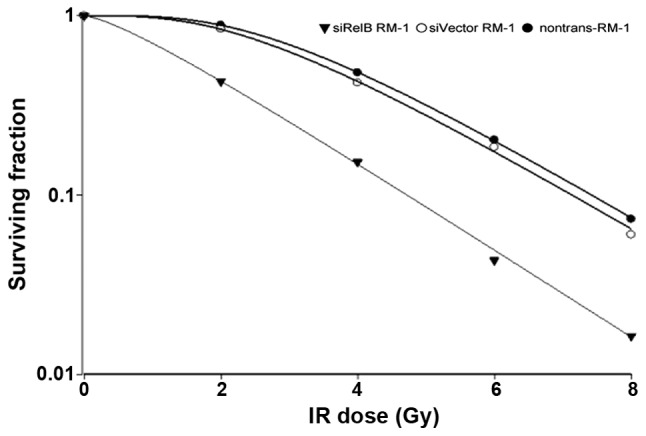
Dose-survival curves of siRelB-RM-1, siVector-RM-1 and non-trans-RM-1 cells following 2, 4, 6 and 8 Gy radiation. Compared with the controls, the siRelB-RM-1 cells were more sensitive to irradiation. ^▼^siRelB-RM-1, pLentilox-sh-RelB-transfected RM-1 cells; ^○^siVector-RM-1, RM-1 cells transfected with empty vector; ^•^non-trans-RM-1, non-transfected RM-1 cells; IR, ionizing radiation; small interfering.

siRelB-RM-1, siVector-RM-1 and non-trans-RM-1 cells were subjected to radiation exposure at 2, 4, 6 and 8 Gy doses. A clonogenic survival assay was performed, as aforementioned. As shown in Fig. 2, the SF values from the siRelB-RM-1 cells (0.8, 0.11, 0.06 and 0.02) were less than that of the siVector-RM-1 cells (0.95, 0.8, 0.12 and 0.09) at the corresponding dosage of 2, 4, 6 and 8 Gy respectively, indicating that the siRelB-RM-1 cells were more sensitive to radiation-induced cell death compared with the controls. Increases in D_0_ indicate higher cell radiation resistance, while D_q_ represents the ability of a cell to recover from sublethal damage. Table I shows that the values of D_0_ (1.68) and D_q_ (0.60) in the siRelB-RM-1 cells were significantly lower than those of the non-trans-RM-1 (1.02, 3.08, 0.89 and 4.97) and siVector-RM-1 (1.93, 2.76, 0.84 and 4.17) cells, indicating that the RelB siRNA-transfected cells had lower radiation resistance and a weakened damage-recovery ability.

### RelB siRNA transfection of RM-1 cells increases radiation-induced apoptosis by inhibiting the expression of the Bcl-xl gene

Once the siRelB-RM-1, siVector-RM-1 and non-trans-RM-1 cells had been exposed to X-rays at a 6 Gy dose, the cells were incubated for another 48 h. These cells were collected along with the RM-1 cells without radiation treatment. Apoptosis was measured using the Annexin V/PI flow cytometry assay, as aforementioned. The results showed that the siRelB-RM-1 cells had a much higher apoptosis rate (15.27±1.62) than the siVector-RM-1 (8.40±0.69) and non-trans-RM-1 (7.90±1.50%) cells.

**Figure 3:**
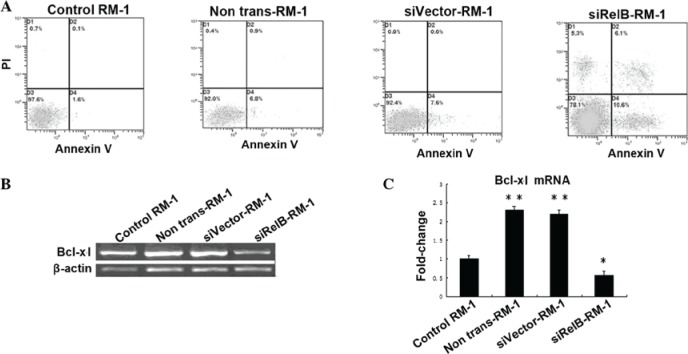
(A) Apoptosis induction with radiation in siRelB-RM-1, siVector-RM-1 and non-trans-RM-1 cells. The apoptosis rate was measured with Annexin V/PI flow cytometry in the various RM-1 cells that were treated with 6 Gy radiation, along with the untreated control RM-1 cells. Radiation treatment induced apoptosis in all the treated cells compared with the control cells. (B) Irradiation activates Bcl-xl in RM-1 cells. The cells were pretreated with siRelB prior to irradiation. The RM-1 cells were subjected to qPCR assay. Total RNA isolated from irradiated cells and untreated control RM-1 cells. (C) Bcl-xl mRNA levels were measured by qPCR assay normalized by the level of β-actin. Significant differences were observed, as indicated, when compared with the untreated groups (^*^P<0.05 and^**^P<0.01 vs. control). si, small interfering; control-RM-1, non-irradiated RM-1 cells; non-trans-RM-1, non-transfected RM-1 cells; siVector-RM-1, RM-1 cells transfected with empty vector; siRelB-RM-1, pLentilox-sh-RelB-transfected RM-1 cells; qPCR, quantitative polymerase chain reaction.

Next, to determine whether the adenovirus-mediated siRNA-targeting of RelB changes the radiosensitivity of PCa cells due to modulation of Bcl-xl gene expression, the levels of mRNA from the Bcl-xl gene was quantified by qPCR (Fig. 3). As shown in Fig. 3, the expression levels of the Bcl-xl mRNA in the non-trans-RM-1 or siVector-RM-1 cells was increased by ~2.3- or 2.2-fold, compared with those in control-RM-1 cells, as observed by qPCR (P<0.05). Consistent with its inhibition of RelB (Fig. 1), siRNA targeting RelB specifically and significantly eliminated irradiation-dependent increases in the levels of the Bcl-xl mRNA in the RM-1 cells. These data showed that adenovirus-mediated siRNA targeting RelB could significantly inhibit the expression of the Bcl-xl gene in the RM-1 cells (Fig. 3). The expression levels of the Bcl-xl mRNA in the siRelB-RM-1 cells were decreased by ~75.3 or 73.1% compared with those of the non-trans-RM-1 or siVector-RM-1 cells (P<0.01; Fig. 3). Overall, these results indicate that inactivation of Bcl-xl using siRNA targeting RelB may be a significant mechanism for the radiosensitization effects of siRNA targeting RelB on the survival of PCa cells.

## Discussion

Despite the fact that the incidence and mortality rate of PCa is low in China, a significant increase was recorded between 2003 and 2007 (13). The mainstay method for the treatment for early-stage PCa includes active surveillance, surgery, external beam radiation therapy and brachytherapy, while the management of more advanced localized disease is generally via a combination of methods and frequently includes the addition of ADT. Radiotherapy is a commonly used treatment for various malignancies and has a prominent role in the care of PCa patients. Attempts to improve the therapeutic ratio of radiation via technological and pharmacological methods has resulted in significant progress in cancer care. Previous studies have indicated that only a minority of patients achieve a complete pathologic response to therapy due to the radioresistance of these tumors, and PCa is no exception (14).

The androgen-independent PCa RM-1 cell line is derived by the transformation of cells from the genital ridge of embryonic C57BL/6 mice with ras and myc oncogenes. Given their characteristic histopathology, RM-1 cells have been considered to be suitable for PCa studies (15). Numerous studies have been undertaken to establish an improved understanding of the mechanisms by which the inhibition of the alternative NF-κB pathway increases radiation sensitivity in PCa (4,16,17). From these experimental data, it was concluded that the RelB-based alternative NF-κB pathway plays a significant role in protecting PCa cells against IR, and that selective inhibition of RelB may be effective for enhancing the susceptibility of PCa cells with high Gleason scores to IR (18). In our previous study, RelB siRNA-expressing lentiviral vectors targeting the RelB gene were conducted with the molecular biological technique to silence the RelB expression in RM-1 cells (6). In the present study, the RM-1 cells were transfected with pLentilox-sh-RelB followed by irradiation, and western blot analysis was subsequently employed to detect the expression of RelB in the cells. It was demonstrated that RelB was overexpressed in the non-trans-RM-1 and siVector-RM-1 cells following IR treatment compared with the control-RM-1 cells without irradiation. Moreover, pLentilox-sh-RelB targeting RelB significantly downregulated the expression of the RelB protein in the siRelB-RM-1 cells following IR treatment.

The development of the resistance of PCa to radiation is a significant complication in treating this disease. Eliminating these radioresistant cancer cells is perhaps the most effective method for decreasing the recurrence of cancer following radiotherapy. The radioresistance of the RM-1 cell line was determined using a colony-forming assay in the present study. Subsequently, it was observed that RelB inhibition by pLentilox-sh-RelB significantly inhibited colony formation in the mouse PCa RM-1 cell line following treatment with pLentilox-sh-RelB for 48 h. In line with the observations of a previous study (18), the present study demonstrated that mouse PCa RM-1 cell SF number and other survival parameters following IR treatment were lower in the siRelB-RM-1 cells compared with the siVector-RM-1 cells. These data indicated that pLentilox-sh-RelB enhanced the radiosensitivity of the RM-1 cells and made them more likely to be killed by radiation treatment.

In the present study, pLentilox-sh-RelB was able to increase the radiosensitivity of the RM-1 cells *in vitro*, which may be mainly associated with apoptosis enhancement. As a consequence, apoptosis may be considered as the most suitable method of anticancer therapy (19). The main aim of apoptoaia-based treatment is to cause tumoral cell death while limiting the cytotoxic effects on healthy tissues. This may be achieved by, for example, promoting the expression of pro-apoptotic factors at the same time as reducing the expression of anti-apoptotic factors in the tumor cells only. The present study found that the apoptosis rate was much higher in the siRelB-RM-1 cells compared with the siVector-RM-1 and non-trans-RM-1 cells. Mineva *et al* (20) demonstrated that RelB-knockdown using siRNA promoted apoptosis in WEHI 231B lymphoma cells, which is accordant with the present results. In the present study, pLentilox-sh-RelB was able to reverse the radioresistance of the RM-1 cells by increasing the level of radiation-induced apoptosis. The level of radiation-induced apoptosis increased to a significant extent, which indicated a significant role for RelB in the control of irradiated PCa cell survival, possibly involving the activation of the anti-apoptotic factors. However, the manner in which RelB was able to affect the apoptosis in the RM-1 cells remains unclear and requires further elucidation. A key mechanism by which NF-κB controls cell survival is through the enhancement of the transcription of various anti-apoptotic genes, including Bcl-xl.

Bcl-xl is an important novel member of the Bcl-2 family, an anti-apoptotic group that has been reported to be vital in tumor progression, development and chemo- or radioresistance (21). Strick *et al* (22) showed that the expression of proteins (Bcl-xl and BAX) from the Bcl-2 family was able to modulate radiosensitivity in human glioma cells. Moreover, Li *et al* (23) proposed that, in order to effectively overcome the acquired radioresistance of cancer cells, the overexpression of Bcl-2 and Bcl-xl may be targeted. Additionally, it has previously been reported that Bcl-xl is overexpressed in PCa and involved in radioresistance, which is also altered by modulating RelB level in cells (17,24). In the present study, following the irradiation of the RM-1 cells, the expression levels of Bcl-xl were relatively high. qPCR analysis indicated that Bcl-xl was expressed in the murine hormone-resistant PCa RM-1 cells and that the expression of Bcl-xl was upregulated in the non-trans-RM-1 and siVector-RM-1 cells following irradiation compared with the control-RM-1 cells without irradiation. Moreover, the expression of Bcl-xl was downregulated in the siRelB-RM-1 cells treated with pLentilox-sh-RelB compared with the siVector-RM-1 and non-trans-RM-1 cells. Overall, the results indicated that IR induces the expression of Bcl-xl in PCa cells to protect the cells against IR, and that RelB-specific siRNA leads to a decrease in the radiation-induced expression of Bcl-xl mRNA. The inhibition of Bcl-xl may participate in the reduction of responses to IR, which could efficiently enhance the efficacy of radiotherapy.

This is the first study to show that pLentilox-sh-RelB downregulates the expression of Bcl-xl in RM-1 PCa cells *in vitro*. The downregulation of Bcl-xl by pLentilox-sh-RelB may at least partly explain its ability to reverse the radioresistance of RM-1 cells. Further studies are required to determine the mechanisms underlying this phenomenon. The present study data indicated that the decreased radioresistance of the RM-1 cells could be attributed to the promotion of apoptosis by the downregulation of Bcl-xl expression, and also revealed the potential benefit of pLentilox-sh-RelB treatment in conjunction with radiotherapy for PCa treatment. In summary, the present results indicate that the alternative NF-κB pathway appears to be important for radiation resistance in PCa cells, and that the inhibition of Bcl-xl with pLentilox-sh-RelB and the promotion of apoptosis may reverse the radioresistance of RM-1 cells *in vitro*.

## Figures and Tables

**Table I:** Values of the varying parameters of the one-hit multi-target model fitted to the non-trans-RM-1, siVector-RM-1 and siRelB-RM-1 cells following radiation treatment.

Cells	D_0_	D_q_	N	SF_2_	SER (D_0_)	SER (D_q_)
Non-trans-RM-1	1.92	3.08	4.97	0.89		
siVector-RM-1	1.93	2.76	4.17	0.84	0.99	1.12
siRelB-RM-1	1.68	0.60	1.43	0.43	1.14	5.13

si, small interfering; non-trans-RM-1 cells, non-transfected RM-1 cells; siVector-RM-1, RM-1 cells transfected with empty vector; siRelB-RM-1, pLentilox-sh-RelB-transfected RM-1 cells; D_0_, mean lethal dose (Gy); D_q_, dose quasithreshold (Gy); N, N-value; SF_2_, 2 Gy survival fraction; SER, sensitization enhancement ratio.
